# ‘He called me out of the blue’: An ethnographic exploration of contrasting temporalities in a social prescribing intervention

**DOI:** 10.1111/1467-9566.13482

**Published:** 2022-05-24

**Authors:** Kate Gibson, Suzanne Moffatt, Tessa M. Pollard

**Affiliations:** ^1^ Faculty of Medical Sciences Population Health Sciences Institute Newcastle University Newcastle upon Tyne Newcastle upon Tyne UK; ^2^ Department of Anthropology Durham University Durham Durham UK

**Keywords:** (a)synchronicity, ethnography, health interventions, linearity, time

## Abstract

Social prescribing, a way of connecting patients to local services, is central to the NHS Personalised Care agenda. This paper employs ethnographic data, generated with 19 participants between November 2018 and July 2020, to explore the socio‐temporal relations shaping their experiences of a local social prescribing intervention. Our focus is on the ways in which the intervention synchronised with the multitude of shifting, complex and often contradictory ‘timespaces’ of our participants. Our focus on the temporal rhythms of everyday practice allows us to trace a tension between the linearity and long horizon of the intervention and the oft contrasting timeframes of participants, sometimes leading to a mismatch that limited the intervention's impact. Further, we observed an interventional ‘drift’ from continuity towards unsupported signposting and ‘out‐of‐the‐blue’ contacts which favour the temporality of the intervention. We demonstrate a need for intervention planning to be flexible to multiple, often conflicting, temporalities. We argue that health interventions must account for the temporal relations lived by the people they seek to support.

## INTRODUCTION

Public health interventions targeting long term conditions (LTCs) build on the identification of linear (although often complex) pathways linking causal factors to behaviours that in turn lead to poor health outcomes (Blue et al., 2021). As Yates‐Doerr (2020) notes, even interventions claiming to target the social determinants of health, such as the social prescribing intervention we discuss here, rely on unidirectional arrows to trace the line of cause (determinants) and effect (health). Thus health interventions which intend to *intervene* in causal pathways and produce new outcomes are formulated according to a linear logic of cause and effect. However, this inherent linearity neglects sociality and the complex and relational ways in which health is made, lived and done. This paper takes the concept of time as its primary concern in order to problematise the linear causal narrative of a local social prescribing intervention.

Aiming to improve health outcomes, in particular for those with LTCs and/or mental health issues, social prescribing interventions are heterogeneous in their approach, focus and duration as well as the populations they target (Husk et al., 2020; Tierney et al., 2020). What these interventions have in common is that they aim to improve physical, social and mental wellbeing (Younan et al., 2020) by providing a non‐medical referral pathway to community or social activities and services, such as benefits advice, fitness classes, and social groups. From ‘signposting’ (providing individuals with information about relevant services) through to models which involve a link worker who interacts with patients to create personalised plans and ‘link’ them to sources of support (NHS England, 2020), social prescribing is understood to address the social determinants of health (Polley et al., 2017). Thus, the rationale underpinning social prescribing deviates from a hegemonic biomedical approach to health, in favour of a more psycho‐social approach to care (Southby & Gamsu, 2018).

At the same time however, social prescribing remains embedded within a healthcare system which is increasingly entrenched in neoliberal discourses of individual responsibility and choice (Mackenzie et al., 2020). For instance, social prescribing is a central part of the UK government's Personalised Care agenda which sets out to grant patients ‘choice and control over the way their care is planned and delivered’ (NHS England, 2019, no page). Yet, as Mol notes ‘good care’ has little to do with increasing patient choice. On the contrary, good care ‘tinkers’ with the conditions in which collectives live (2008, p. 68). Through the contrasting logics of choice and care, Mol argues that the foregrounding of individual choice in healthcare settings is often at the expense of care. Similarly, Hirvonen and Husso (2012) note that the increasing economic‐rationalist compression of public sector time runs counter to the relational understanding of time inherent in care. Thus, social prescribing is particularly interesting because while it represents an attempt to reconcile the apparently oppositional dichotomy of choice and care through offering individually tailored support, interventions are nevertheless steeped in prevailing ideas of ‘personal responsibility, choice, and [therefore] blame’ partly maintained by bureaucratic temporal structures (Blue et al., 2021, p. 1052).

The social prescribing intervention we discuss here targets individuals with LTCs living in an ethnically and socially diverse urban area of North East England, many of whom live in marginalised communities. Link workers engage with ‘clients’ to help them identify personalised action plans and work towards goals. With some clients remaining with the intervention for up to 4 years, the intervention aims to improve clients' health and wellbeing over a lengthy duration via prolonged contact between link workers and clients. This paper explores clients' experiences of this local social prescribing intervention via the temporal vantage point offered by an ethnographic lens.

## TIME

We have previously reported on how class shapes clients' engagement in social prescribing, specifically as it relates to the future‐focussed temporal orientations of health interventions (Gibson et al., 2021). In this paper, our focus is on clients' multiple and shifting temporal experiences, and in particular, how these relate to an intervention‐focussed view of time as fixed, constant and linear (timeline).

To conceptualise clients' experience of time as it relates to the delivery of a local health intervention, our focus is on the everyday organisation of practices (including practices of engagement) which occur in multiple, fluid, and intersecting dimensions of time. In this framing, temporal rhythms ‘both create and respond to practitioners’ experiences of time’ (Blue, 2019, p. 933) and time is a space where practices intersect, sometimes in harmony, sometimes in conflict (Schatzki, 2009). Conceptualising time as non‐linear enables us to see beyond the (often) lengthy duration of this intervention and focus instead on the ‘timespaces’ within which everyday practices are coordinated and aggregated (Schatzki, 2009). In order to achieve this, we take the concept of (a)synchronicity (Brose, 2004; Southerton, 2006) as our primary focus to explore how effective practices of care via social prescribing require the coordination of often conflicting ‘time‐space paths’ (Brannen et al., 2013, p. 418). Following Andersen and Bengtsson (2019) we consider the challenges involved with synchronising bureaucratic time with the rhythms of everyday lives.

Critiques levied at the assumed linear temporality of health interventions are not new. Focussing on intervention delivery, Tufte and Dahl (2016) explore how care workers reconcile temporal dilemmas to negotiate standardised temporal schedules which allocate caring tasks as linear actions. Similarly, Hirvonen and Husso (2012) note that welfare workers are constrained by an economic‐rationalistic framing of time which runs counter to a relational understanding of care which in turn produces an experience of ‘working on a knife's edge’. Nevertheless, the subjective time dimensions of care remain relatively understudied (Andersen & Bengtsson, 2019) and even less is known about how those in receipt of ‘personalised care’ experience ‘intervention time’. Andersen and Bengtsson (2019) explore the experiences of both vulnerable young people and welfare state professionals through the concept of ‘timely care’. They note that ‘*timely care*, defined as the right care at the right time’ requires the temporal rhythms of welfare bureaucracy and young people's needs to be ‘in sync’ (2019, p. 1510). They argue that in order to identify why ‘bureaucracies routinely fail to provide timely care’, we need a deeper understanding of subjective time dimensions of care (ibid., p. 1511). That is, accounting for the experiences of those receiving care allows us to explore the relationship between interventional effectiveness and timing.

McCoy's examination of the work involved in medication adherence is particularly useful in this respect because it centres analysis on the ‘embodied, purposeful, skilled’ (2009, p. 132) work done to achieve a three‐way alignment between clock time, inner experience and the requirements of a medication schedule. That is, doing adherence, and as we would argue, doing health more generally, entails a form of ‘time work’ to create a temporal space at the junction of subjective time and objective time. Partly inspired by Flaherty's (2003) concept of time work – the work individuals undertake to manage temporal experiences – McCoy opens up the concept to account for ‘anything people do, deliberately and with some acquired skill, that in some way orients to time’ (McCoy, 2009, p. 131). Importantly, time work is ‘conditioned by the social context in which it arises’ (Flaherty, 2003, p. 30). Thus, the concept of time work allows us to understand client temporal experience as something which is not passively experienced but actively realised and mediated in relation to objective constraints which themselves shape the possibilities for engaging with a health intervention. Moreover, paying ethnographic attention to the ‘time work of aligning individual lived experience with standardised clock time’ (McCoy, 2009, p. 130) highlights the shifting tensions between ‘intervention time’ and subjective experiences of time. Indeed, as Andersen and Bengtsson note ‘the need for care is not fixed in time and space’ (2019, p. 1522).

In what follows, we demonstrate that the ability of the intervention to deliver ‘timely care’ (Andersen & Bengtsson, 2019) rests on an intricate and nuanced relationship between the constraints of ‘intervention time’ and the temporal rhythms of daily life. In so doing, we argue that the linear organisation of care delivery leaves the relationality of doing health unattended, and more specifically, we show the effects of that on the people the intervention is designed to care for. We first outline the methods and situate the value of ethnographic research for understanding the multiple temporal rhythms shaping engagement. Second, we share our participants' highly varied temporal experiences and draw attention to the socio‐temporal relationships shaping what people are able to ‘do’ with their social prescribing time. Third, we turn our attention to the ways in which the intervention connected with our participants' lived realities. We find a tension between an intervention‐organised timeline and the timespaces of those it seeks to support. While some participants experienced an embedded form of social prescribing which synchronised with and was reactive to the everyday rhythms of their lives, others experienced a form of social prescribing which was constrained by linearity and therefore asynchronous with their lives. We conclude the paper by discussing the value of health interventions which sufficiently anchor into clients' temporalities and are flexible to the multiple power relations shaping their engagement.

## METHODS

The ethnography we present here unfolds along the temporal rhythms of ‘people time’ rather than the linearity of ‘project time’ (Koster, 2019). Foregrounding client temporal rhythms allowed us to capture the nature of interventional synchronicity thus allowing for an intimate understanding about *how* the intervention connects with clients. The ethnography is part of a larger, mixed methods evaluation of the social prescribing intervention (Moffatt et al., 2022). It contributes to the small, but growing, research body which uses ethnography in intervention research (Hughes, 2019; Lewis & Russell, 2011; Williams & Fullagar, 2019) as a means to explore how interventions ‘show up’ in settings (Hawe et al., 2009).

Overall, KG spent 200 hours over 20 months with participants and/or their families in various degrees of intensity depending on their preference. Some participants were visited regularly and contacted by phone at least once a week; for others ethnographic encounters were more ad hoc and infrequent. Participant observation ranged from visiting homes, meeting in coffee shops, and travelling by bus or by foot to services and activities such as fitness classes, debt advice, walking groups, social groups, gardening, the foodbank, and attending link worker meetings. By ‘being there’ we aimed ‘to experience the mundane and sacred, brash and nuanced aspects of socio‐cultural life and, through observations, encounters and conversations, to come to an understanding of it’ (Lewis & Russell, 2011, p. 400). It is important to note that even for those with whom KG spent extensive periods of time, the ethnography represents a small and partial connection with participants' lives (Hughes, 2019). To some extent, our ways of finding out mirrored the intervention, not in terms of our fieldwork's longitudinal nature but in the way that our timespace paths connected with those of our participants, or not. Our ethnographic insights are derived from the piecing together of such moments of synchronicity. While these snapshots result in an ‘interrupted conversation’ (Reynolds & Lewis, 2019, p. 11), we have been continuously in tune ‘to the gaps and the in‐between spaces of the ethnographic encounter, and therefore, in turn, to the gaps and in‐between spaces of how an intervention unfolds and brings about change (or otherwise)’ (ibid., p. 11). There were many moments in this research which offered opportunities for reflection in what Mannay and Morgan (2015) refer to as the ‘waiting field’. Rather than interrupting the generation of data, these ‘in‐between spaces’ offered further vantage points from which to engage deeply with participants' everyday experiences. For instance, sometimes participants were late because they relied on public transport, and they cancelled meetings because they needed to work extra shifts or the icy weather made walking with limited mobility too risky for them to leave the house. These ‘spaces previous to’ (ibid., p. 174), times of waiting and uncertainty, engendered opportunities to engage deeply with participant time because we experienced how different priorities and requirements shaped and interrupted their everyday lives. Hence, while the ethnographic enquiry which informs this paper was conducted over 20 months, its value lies not in its promise of telling a complete story, but rather in its capacity to tell a temporally inflected story based on a rich empathetic understanding of our participants' social worlds.

The ethnography was conducted with a sample of 19 participants. As with all clients in this intervention, they were aged between 40 and 74, and had at least one of eight LTCs (diabetes type 1 and 2, chronic obstructive pulmonary disease, asthma, coronary heart disease, heart failure, epilepsy and osteoporosis with or without anxiety and/or depression). During recruitment, our sample was continuously monitored for age, gender, ethnicity, length of time with the intervention, and residential and employment status. We aimed to capture a diverse range of people, all at varying points in their social prescribing journey whilst fulfilling the wider project's eligibility criteria that participants had type 2 diabetes (T2D). Nine participants had been referred into the intervention during its early stages (September 2015‐September 2017) and were discharged, or close to discharge, during the ethnography. The remainder of the sample were referred into the intervention just prior to or during fieldwork (January 2018‐March 2019) and were understood to be in ‘active engagement’. In addition to participant observation, fieldwork entailed an initial semi‐structured interview (*n* = 19), exit interviews (*n* = 15), photo‐elicitation interviews (*n* = 9), interviews with family members (*n* = 7), and data documenting client journeys (for example, notes made by link workers following client meetings) were provided by the intervention with participants' consent (*n* = 15). Interviews were audio‐recorded and anonymised during transcription. Fieldwork was conducted in person and face‐to‐face until March 2020, from which date all fieldwork, including exit interviews was conducted over the phone or by video‐call due to Covid‐19 restrictions. Due to KG's extended relationship with the participants, our use of remote methods during the pandemic had little negative impact on data collection. Indeed, KG's continued contact during the first wave of Covid‐19 was valued by many participants, many of whom were significantly affected by lockdown restrictions as we detail elsewhere (Morris et al., 2022). Durham University Research Ethics and Data Protection Committee provided ethical approval for the research.

Analysis was a continuous process throughout data collection and largely informed by reflexive field notes written directly after each research encounter. On completion of fieldwork, the dataset, comprising interview transcripts, researcher and participant photos and field notes, was analysed in its entirety through a process of full immersion with the assistance of NVivo 10 which was used to code, sort and store the data. Data were analysed in an iterative and inductive process of constant comparison and recoding of the data in order to capture points of significance, conceptual or otherwise (Glaser & Strauss, 1967). This process was achieved collaboratively across the team (KG, SM, and TP), generating a range of insights pertaining to the timing of social prescribing as what follows will explore.

## SUBJECTIVE EXPERIENCES OF TIME

In this section, we consider three interrelated aspects of people time: temporal ruptures, harriedness, and waiting. As what follows will show, paying attention to our participants' experiences of such temporal flows lays bare the material and social constraints under which engagement with this social prescribing intervention occurs.

### Temporal ruptures: ‘something else creeps in and knocks you right down’

The everyday rhythms of our participants' daily lives had multiple temporal features and yet as Zaheer emphasises below, such shifting temporalities were often absent from participants' narratives relating to their health and the intervention:

When you see people just moving along at their own pace it is telling me I don't need to rush into anything, just go at my own pace and develop my own steps to change myself rather than being forced to change or whatever. It's giving me encouragement.

Zaheer describes his understanding that ‘changing himself’ is achieved by moving along at a slow and steady tempo inspired by others. Brose argues, ‘a linear time awareness acquires the function of bridging the ruptures in order to construct a continuity’ (2004, p. 14). However, Zaheer's construction of a steady linear progression, which gives him ‘encouragement’, is in sharp contrast to his comment elsewhere in this interview:

Health is up and down, I try to put a brave face but sometimes financial side, sometimes the domestic side… it's linked and sometimes just moves in at the wrong time and as soon as you have a little bit of strength in yourself and you are starting to manage something else creeps in and knocks you right down again.

Here we learn that the ebbs and flows of institutional and structural processes interrupt Zaheer's account of ‘moving along’ his linear (albeit slow and steady) journey of self‐improvement. As he suggests, finances and familial relationships intersect with the spatio‐temporalities of his health, often ‘moving in at the wrong time’. Contrary to his ideal, like all participants, Zaheer's lived experience of time is beset by ‘twists and turns’ (Mol, 2008, p. 44).

Across the sample, participants experienced temporal ruptures associated with for instance, bereavements, housing, changes in employment, and health challenges, often bringing a nexus of interrelated practices into tension. For example, the hospitalisation of Jill's ageing parents and subsequent death of her father made following her weekly ‘healthy’ menu untenable:

With being at the hospital most days, the menu that we tend to do has gone out of the wall. However, we are cooking tonight what we should be doing. But we had a Chinese on Friday, because it was y'on time [late] when we got back.

Our participants' experiences of temporal ruptures bring into focus structural conditions such as class, gender, and ethnicity. Previous research has documented that access to resources can minimise the lasting impacts of temporal ruptures (O’Donnell, 2020). Similarly, Jill who owned her own home with her supportive husband and was in secure employment, soon returned to her weekly menu once her mother had settled into a nursing home. Elsewhere, a chest infection and the side effects of changing T2D medications made Labani, a south Asian migrant, ‘*feel dizzy and have diarrhoea’* meaning that she was unable to attend her local college where she was required to learn English in order to claim state benefits. Labani, aware of the impending temporal rupture non‐attendance at the class would cause, contacted her doctor fortnightly to verify her ill‐health. She explained, ‘*I don't show a sick note then they cut the benefit’*. A few months later, Labani and her family visited family in South Asia, a common practice amongst transnational families (Mand, 2010). This itself culminated in another temporal rupture; her family's benefits were sanctioned because they had left the country for several weeks.^1^ Like many participants in receipt of state welfare, the instability of living with limited economic resources required that they borrow money *‘from here and there’*. Evidently, the social relations which facilitated the re‐establishment of Jill's routinised healthy eating practices left Labani vulnerable to temporal ruptures and struggling to mitigate their effects.

As our examples thus far suggest, doing health occurs in a net of interwoven (and often conflicting) timespaces (Schatzki, 2009) which magnify (and are magnified by) objective power relations. We now focus on the contrasting temporalities of harriedness (Southerton, 2003) and waiting, which both emerged as significant temporal experiences in our dataset. For Southerton (2003), harriedness is generated by a need to schedule activities within otherwise compressed timeframes. While at face value, waiting seems antonymous with harriedness, waiting also requires a significant investment of time: the requirement to fill otherwise ‘empty’ time (Flaherty, 2003). What ties both timespaces together is an analytical frame which conceptualises experiences of time as realised through practices which are responses to socio‐temporal structures. Our focus on waiting and harriedness therefore enables us to consider how participants were able to find time to engage with the intervention.

### Harriedness: ‘I'm tired. I don't have time for myself’

Some of our participants negotiated conflicting timespaces comprised of interdependent responsibilities, the successful coordination of which related to their temporal autonomy (or lack of). Take Bobby, for example: a working‐class man who worked as a night‐time security guard and lived with and cared for his elderly mother. When KG visited Bobby towards the end of fieldwork, she learnt that his doctor had diagnosed him with high blood pressure and, because he had gained weight, suggested he attend a diet management course. He stopped going after the first session, citing paid employment as the reason: *‘what can you do? I get paid for extra shifts so I can't miss them’*, he said. Warren's (2003) intersectional analysis of the classed and gendered processes which constrain temporal autonomy found that ‘time poverty’ created by shift work (a regular feature of working‐class employment) impacts on families achieving shared time schedules. Similarly, Bobby's shift work did not synchronise with the nutrition class. Given his economic need to work extra shifts, Bobby prioritises fulfilling his paid work commitments which are temporally incompatible with attending the class.

Unsurprisingly, many female participants occupied temporal spaces which were intersected with caring responsibilities for family members as Jill's example above suggests. Here, other‐orientated caring responsibilities consistently took priority over the self‐orientated care implied by participating in the intervention. For example, Brenda's commitments to caring for her daughter as she underwent cancer treatment were incompatible with attending an exercise class. Similarly, Carol left the area to care for her ill father who lived elsewhere in the country. For Carol, the interruption culminated in a loss of momentum entirely and she never returned to her exercise sessions as we will explore below. Particularly indicative of this gendered dynamic is Aisha. Originally from Sub‐Saharan Africa, Aisha discontinued her degree when she arrived in England, partly because of language barriers and partly because there was *'too much violence*' from her first husband. She lives in social housing with her four children and second husband, who although currently unemployed cannot claim any form of state welfare because he is not a British citizen. Aisha has significant caring responsibilities for her children, each of whom has a learning disability and/or a LTC. Aisha repeatedly described her need to juggle the competing demands of her caring responsibilities, leaving her too exhausted to prioritise self‐care:

Sometimes I don't take medication because I forgot because I'm so concentrate on the boys… …the last thing I want is somebody to say, ‘You want to see me?’ or appointment or I need to come to school, you know (laughs)? Even to cook. I look. It's not like I feel cook in the way I do. I wait until afternoon and I call the pizza, ‘Can you please deliver please?’ I feel really bad because I'm down, down, down, yes. Yes, it affects a lot if I'm not feeling…

Amid the harried tempo of her everyday life, Aisha opts for the convenience of a takeaway pizza. At face value, an efficient solution to the pressures of her conflicting temporal routines, she nonetheless feels ‘bad’ for not cooking, a culturally recognised means of caregiving (DeVault, 1991). Southerton (2003) argues that avoiding harriedness is dependent on the amount of control individuals have to synchronise conflicting schedules. Research establishes that women are disproportionally affected by time constraints associated with domestic caring responsibilities and often responsible for synchronising often irreconcilable spheres of their lives with those of their family members (Brannen et al., 2013). Therefore, it is unsurprising that Aisha's (and many women living in similar circumstances), other‐orientated care responsibilities leave little time for self‐care – indeed she barely has time to achieve her other‐orientated care.

Aisha had been linked into a series of in‐house counselling sessions, attendance at which she valued, although she missed a few appointments due to her children's more pressing health concerns. An important point to make here is that Aisha (and crucially, her counsellor) understand her otherwise all‐consuming time constraints as something which can be simply overcome by ‘*giving myself time*’.

Now I need to concentrate on something for myself, give myself time. You know, I give myself time, one of the things she [the counsellor at the intervention] advised me there. When's the last time you called one of your friends? Just call, ask. This is a way to start.

We never learn whether Aisha manages to ‘give herself time’. Just as her caring responsibilities required her to miss several counselling sessions, so too did they take priority over her participation in the ongoing ethnography. Spotswood et al. (2021) find that the temporal features of motherhood shape the possibilities of engaging in self‐orientated care. Likewise, Aisha's time is relational and intricately connected to both the temporal routines of caring for her family and the sociocultural conditions within which they occur. Similarly to Labani, as an economically marginalised migrant mother, race, class and gender intersect to position her as ‘structurally vulnerable’ (Isaacs et al., 2020). Thus, ‘giving herself time’ is not straightforward. Rather, ‘giving herself time’ requires undertaking significant ‘time work’ (Flaherty, 2003) to disentangle herself from a temporal experience of harriedness as well as ‘self work’ (McCoy, 2009) to prioritise creating a timespace in which she can pursue self‐care. This work is undertaken in relation to dominant versions of (classed) femininity which require her for example, to ‘*feel bad*’ for not cooking, despite her desire to do differently. Hence, caring for herself requires more than having enough time in the quantitative sense. Temporal rhythms must be understood in relation to wider objective constraints which shape what people such as Aisha ‘do’ with their time, even when time is seemingly abundant, as what follows will show.

### Waiting: ‘We've just got to sit and wait’

Several participants experienced apparently empty time‐space zones. For instance, Steve's wife, Pat describes him ‘doing nothing’:

My words are he's just cabbaging. He gets up… …He comes downstairs and his tablets are on the bench ready for him to take and a teabag in the cup. He'll go in the garden, has a smoke and then he'll come through to the living room, puts the news on and that's him set for the day and that's it. That's every day.

Steve has multiple LTCs, significant mental health issues and rarely leaves the house. But while Steve's time is spent ‘cabbaging’, it is not that he is doing nothing. Much of Steve's time is spent *waiting*, for instance for the latest in a series of talking therapies referrals to come into effect or to relocate to more appropriate social housing about which he said he felt ‘*on edge*. *Panicking in case we miss anything*. *Panicking in case we don't get one*.’

Waiting shaped many participants' temporal rhythms, in particular those with limited resources. The effect of endless waiting engenders fatigue (Auyero, 2011), anxiety and frustration (Jowsey et al., 2016). As the example of Carol below illustrates, several participants endured lengthy waiting lists for mental health services, exercise programmes, medical procedures and appointments.

I am just waiting. Just waiting to see what they are going to do, what they are going to say, and when I can have the operation. Because my hernia has just doubled in size. Even though I am losing the weight, that seems to just be getting bigger.

Schwartz notes that ‘waiting subserves the distribution of power that it presupposes’ (Schwartz, 1974, p. 857). Similarly, Carol's experience of waiting underlines her powerlessness: by ‘just waiting’, she is suspended in time. She has no choice but to ‘just’ wait for somebody else to decide what ‘they’ will do with her body, thus undermining her temporal autonomy. Elsewhere, many participants were waiting for state welfare payments, decisions and appeals, and other official, often life‐changing, decisions. For instance, Anna appeared to spend the entire ethnography awaiting the results of her benefits appeal.^2^ Auyero (2011) documents the frustration and tedium inherent in welfare recipients' experience of waiting. Anna, like several others who had limited economic capital, voiced similar experiences: ‘*W* *e've just got to sit and wait’* she said during our final interaction. Just like the harried of the sample, the magnitude of these experiences of waiting and uncertainty often rendered health matters irrelevant. For instance, KG met with Anna when she was awaiting news of her grandchildren's removal into care:

Her daughter is in court today waiting to see if they [social services] are going to take her kids away. When I return with the tea, I learn about what has happened…

…Our conversation is continuously punctured by her checking her phone for news about the court case… …Each time she checks her phone, she gets more and more anxious…

…It's uncomfortable place to be and asking her about anything mundane like her health or everyday life just feels insensitive. Yet in a moment where she is breathless, I ask about her health, despite it feeling completely irrelevant. I learn that her T2D is terrible as is her asthma. She started smoking again and had ’20 tabs [cigarettes] on Monday because of the stress of everything’

[Fieldnotes]

In addition to highlighting the frustration and anxiety magnified by waiting, these fieldnotes are an important insight into the context of waiting experienced by many participants who had no other choice but to wait. As Bourdieu notes (2000, p. 228) ‘waiting implies submission: the interested aiming at something greatly desired durably – that is to say, for the whole duration of the expectancy – modifies the behaviour of the person who ‘‘hangs’’, as we say, on the awaited decision’. Waiting for this highly significant news continuously interrupts our conversation in the same way that waiting shapes the context from which engagement with the intervention could occur. Matters of health are relevant only inasmuch as they are exacerbated by and relate to the context of waiting. Accordingly, smoking is understood not in health‐terms but as an opportunity to relieve the stress of a time rhythm shaped by waiting: to ‘disrupt the dragging of time’ (Warin et al., 2015, p. 312).

The experience of waiting emphasises the link between power and time (Bourdieu, 2000; Schwartz, 1974), just as the experience of harriedness and temporal ruptures are compounded by social constraints. Having set out some of the material and social constraints which shape the experience of time, we now shift our focus to consider how the time*line* of the intervention coordinated with the time*spaces* of participants.

## INTERVENTION TIME

We note above that the social prescribing intervention aimed to deliver holistic, personalised care over a lengthy duration. The intervention was nevertheless underpinned by market logics. Launched in 2015, it was financed through an outcomes‐based Social Impact Bond (SIB), a funding model developed to aid the funding of public services by financially rewarding providers and investors for achieving performance targets (Dayson et al., 2020). The SIB model increasingly incentivised maximising referrals and continued engagement with the intervention following a restructuring of the way in which providers were paid. An outcomes tool served the dual purpose of capturing clients' health and wellbeing progress as well as act as a payment trigger for the providers delivering the intervention. This was completed between clients and their link worker approximately every 6 months. The frequency and nature of contact aside from these encounters varied considerably as what follows will explore.

### ‘She was lovely to talk to’

Some participants, especially those engaged with the intervention in its fledgling years experienced a form of social prescribing which neatly synchronised with their needs. Illustrative of this experience is Masood (referred‐July‐2015) who was living with multiple LTCs and in a volatile domestic relationship. Masood experienced frequent and sustained encounters with his link worker who facilitated a range of support concerning his health and social care needs. For instance, after expressing an interest in learning how to ride a bike, Masood was supported to attend a one‐to‐one bike session. When on the day of the session he was hesitant to attend, his link worker accompanied him to the session. Ultimately, Masood was not comfortable with cycling. A few weeks later, the link worker put in motion a referral to the local gym and once again accompanied him to his first session. Thereafter, the link worker telephoned Masood to remind him about his weekly gym sessions. This ‘persistent tinkering’ (Mol, Moser and Pols, 2010, p. 14) with the complexities and shifting tensions of Masood's social world was highly valued by Masood who described his link worker as ‘*a friend’*. Christine (referred‐January‐2016) also described her link worker as ‘*lovely to talk to*’ and recalled how they met ‘*to have a cup of coffee and stuff*’. Christine was supported to access benefits and housing and over time, established a strong rapport with her link worker:

Just gradually telling her how things are within here and outside and the family. Because I had a few problems with the family at the time.

Likewise, Brenda (referred‐December‐2015) described how her ‘*really lovely*’ link worker had ‘*really looked into everything*’. She recalled their frequent meetings throughout her exercise referral:

He [link worker] used to ask all about it [the gym], and how it made you feel and what you thought about this, and would you recommend it.

Brenda's recollections suggest that exploring the extent to which the gym coincided with Brenda's circumstances was of key concern for the link worker. Furthermore, just as Christine indicates that she ‘*gradually*’ shared details about her family difficulties with her link worker, Brenda's link worker ‘*used to ask all about’* the gym. That is, this kind of support was continuous and intentional rather than a one‐off occurrence. It is well recognised that a strong and supportive relationship with a link worker is integral to the workings of social prescribing (Wildman et al., 2019) and elsewhere, in their study of homelessness, Davidson et al. note the importance of services ‘being there’ and gradually building up trust and rapport (2021, p. 8). As Christine suggests, building trust can take time, and moreover, it requires synergy with client temporalities.

These experiences of ‘timely care’ (Andersen & Bengtsson, 2019) were relatively absent from our participants more recently engaged with the intervention, although it is important to add that when such embedded encounters did occur they were highly effective. For example, Sandeep (referred‐January‐2018) was a shift worker and had been unable to pursue any activities suggested by his link worker, such as attending a local yoga group. He nevertheless appreciated his link worker checking in with him over the phone. He explained:

‘I feel like a friend, when he talks. I don't normally talk to any strangers about how I'm feeling’… …I feel that someone is taking notice and asking me.

During fieldwork, Sandeep's circumstances changed and he was no longer able to work. Because the link worker had continuously ‘*taken notice*’ and sufficiently synchronised with Sandeep's competing responsibilities, Sandeep's change in circumstance was identified relatively quickly by the intervention, and his link worker supported him with several referrals intended to ameliorate his situation.

Otherwise, interventional practices of engagement appeared to have undergone a change in tempo and periodicity over the years. This made it difficult for the temporal rhythms of the intervention to synchronise with those of its clients. A temporal drift was evident, in line with the ‘citizen shift’ identified by Williams and Fullagar (2019), effectively shifting responsibility onto individuals to engage with social prescribing without recourse to context. While the drift itself is not our primary focus here, it is worth noting that this change demonstrates that interventions can themselves be subject to alienating temporal relations. Just as Barnes et al. (2003) argue that short‐term political imperatives to demonstrate ‘quick wins’ can refocus health interventions which originally sought to be long‐term, the intervention we report on here appeared to experience increasing pressures that limited its capacity to invest the time required by some clients. As the following section will demonstrate, a central feature of this apparent drift was that the support which had been previously reactive to and in synergy with the care needs of its clients, became increasingly constrained by the inherent linearity of intervention time. Seemingly a drift to the logic of choice occurred, which we argue was at the expense of care. Scheduling of link worker‐client connections was increasingly dominated by the outcomes tool which structured the intervention's timeline. For some participants, periodic progress reviews entirely dominated their experience of the intervention; the intervention interrupted rather than coincided with their temporalities; and, contact from the intervention was often surprising, or ‘*out of the blue’* as Eddie described. Of particular importance is that our participants were often given responsibility for pursuing their own care. They were often ‘signposted’ without support to activities, and responsible for scheduling engagement with the intervention for any contacts asides from intervention‐scheduled six‐monthly progress reviews. Consequently, the intervention could be out of touch with the ever‐changing nature of many of our participants' everyday lives, many of whom were not accustomed to proactively seeking out support, as what follows will explore.

### ‘How on Earth do you ring someone and say, I can't cope?’

Most participants were aware that they could contact the intervention if they needed anything; as Bobby (referred‐July‐2018) indicated, ‘*if I've got any problems*, *I can always give [link worker] a ring*’. However, Bobby did not update the link worker about his non‐attendance at the nutrition class mentioned above. Likewise, we note above that the weight of Steve's (referred‐November‐2018) worries left him ‘cabbaging’ and completely at odds with the linear, future‐focussed temporal horizon of the intervention. He too did not contact the link worker, whom he had only met once for his progress review, to ask for support about his housing issues.

Similarly, the structured timeline of the intervention was asynchronous with Labani's (referred‐November‐2018) changing financial circumstances following the benefits sanction we detail above. This was even after Labani contacted her link worker. When KG visited her shortly after she learnt that the link worker had conducted a progress review with Labani:

I find out that Labani has been to the GP surgery to meet with the link worker. The link worker had asked about her health and Labani had asked about benefits. I ask if they received help with benefits and she gets out the contents of an envelope for me to look at. I notice a compliments slip which reads, ‘As requested, please find enclosed information…’ Labani shows me two A4 printed pages… …on learning that they do not know what to do with the information, I explain to them that ‘Welfare Rights’ refers to benefits advice.

Contrary to the earlier experiences of Masood, Brenda, and Christine, Labani's link worker did not revisit Labani's benefits issues and Labani did not contact the link worker again, even when her benefits were sanctioned. Consequently, like Bobby and Steve, her difficulties went unnoticed, and therefore unsupported, by her link worker.

In addition to a risk that temporal ruptures would inevitably be unnoticed when interventional engagement is constrained by fixed and linear temporalities, placing onus on clients to ‘make room’ for engagement could further perpetuate the alienating and uncertain temporal relations already dominating some participants' lives. For example, Carol (referred‐March‐2019) was finding it difficult to engage with the intervention. As we indicate above, caring for her father had interrupted her attendance at the local gym, making it hard to return. But Carol was reluctant to contact her link worker even though she felt her link worker had been supportive, having ‘*opened up a whole new world for me’*:

a couple of times I've wanted to ring her, because I've been in such a state and then I've sat back and thought, no I can't, she might be with a client. No, I can't, you know, how on Earth do you ring someone and say, I can't cope. And then I feel stupid.

Carol was discharged from the intervention due to ‘lost engagement’, and after a series of cancellations, KG also lost touch with Carol. A year later Carol participated in an exit interview thus offering a rare glimpse into her experience following ‘non‐engagement’. Carol reflected back over her short experience of the intervention, explaining that she had begun to avoid the link worker's calls. During the interview, she became extremely upset recalling how the intervention ‘*gave up*’ trying to contact her and rebuked herself for not contacting her link worker:

I think she just gave up trying to contact me and that kind of thing. I ignored the phone. I didn't answer any of the letters. I didn't contact her to say that I was struggling, which is what I now know I should have done. I should have been able to pick up that phone and say to her, ‘[link worker], I am really not coping, and I mean I am really not coping.’ I should have let people in to tell them how rough I felt, how at one point suicidal I felt.

Carol chastises herself for not prioritising contacting the link worker, despite indicating that she wanted to call her. She said later in the interview, ‘*it was nothing to do with them*. *It was my fault’*. Arguably her inclination to ask for support was shaped by an individualised self‐understanding that she ‘*should*’ have done better. As she said in her first interview, ‘*I just need her because I'm not dealing with things very well*.’ As Mol (2008) argues, those who feel guilty are unlikely to engage in self‐care such as contacting her link worker for support.

We have thus far shown some of the ways in which pre‐defined linear sequencing impeded the provision of ‘timely care’ (Andersen & Bengtsson, 2019). Furthermore, we note that the coordination and alignment of time rhythms required for engaging with the intervention and its linked support increasingly became our participants' responsibility. For many of our participants, the responsibility for synchronising often irreconcilable timespace paths was difficult, and sometimes impossible, to achieve. This is a fundamental shortcoming of health interventions built on assumptions about choice (NHS England, 2019). Allocating responsibility to people to actively connect their time with the unilinear temporalities of interventional time has consequences. As Carol indicates, a lack of connection becomes positioned as a moral failing, rather than the outcome of asynchronous temporal rhythms.

## DISCUSSION

A number of studies have demonstrated the importance of accounting for context in the evaluation of interventions (Hawe et al., 2009; Orton et al., 2017) but few have accounted for context by focussing on the temporal rhythms shaping everyday practice. In this paper, we have emphasised that engaging with a local health intervention, or ‘doing’ health more broadly, is inextricably connected to multiple and diverse temporal relations. We have drawn attention to the shifting tempos and temporal boundaries characterising our participants' everyday lives where self‐care practices (such as engagement in social prescribing) occur within a nexus of often conflicting pressures such as negotiating state benefits, shift work, and family responsibilities. These constellations and bundles of everyday responsibilities are embodied and reproduced (Blue et al., 2021) to create a range of temporal patterns, which are often in conflict with the linearity of intervention time. In light of this, we emphasise the importance of health interventions synchronising with clients' temporal rhythms as well as prioritising care which ‘tinkers’ with the objective constraints shaping clients' temporal autonomy.

The observed disconnections between the intervention and many of our participants emphasise some of the issues with delivering care according to a model which is underpinned by a unilinear timeline. We find that as intervention time became constrained by the linearity of the logic of choice, many participants were unable to coordinate their time‐space paths with those of the intervention meaning that their difficulties were unseen by the intervention. A consequence of the limited capacity and timescale of the intervention was the allocation of personal responsibility to clients for incorporating and pursuing social prescribing. However, some participants struggled to integrate social prescribing into timeframes which were often beset with temporal ruptures and competing priorities, often leading, as we have shown, to either a harriedness or fixed sense of waiting that did not allow for engagement with social prescribing. There are consequences of positioning the active engagement with social prescribing or other forms of personalised interventions as a choice. The requirement to individually pursue care not only creates a significant ‘time burden’ to participants (Jowsey et al., 2016), but also people who ‘fail’ to ‘*just* give themselves time’ (italics added) as Aisha suggests, or proactively contact the intervention (as indicated by Carol's remark that she ‘should’ have contacted her link worker) risk being characterised as individually failing both by themselves and the intervention intended to support them within the social contexts of their lives. Thus, not only does the intervention fail to offer them the support they require, but it may actively cause harm by creating stigma.

The experiences we present here are not representative of all clients' experiences of social prescribing, nor is this intervention representative of all social prescribing interventions. However, in offering this situated critique our scope is broad. For instance, our inclusion of valued interventional experiences, such as those of Masood, Christine, Brenda, and Sandeep, highlight what embedded and synchronised ‘timely care’ (Andersen & Bengtsson, 2019) can achieve when it is not rigidly bound by linear progression or constrained by time scarcity. Here we found the intervention favoured delivering care which tinkered with our participants' competing practices and priorities, so as to ensure that their engagement in social prescribing was both resilient to and a source of support during possible temporal disruptions. Hence, we suggest that the temporal organisation of health interventions, such as the social prescribing intervention we report on here, must be malleable to people's temporal rhythms and the oft‐competing lived realities shaping their engagement. Echoing Yates‐Doerr, we suggest that local health intervention design ‘doesn't act from afar in a linear direction, but acts by engaging, listening to, and adjusting itself in response to how it is taken up by the people whose lives it seeks to impact’ (2020, p. 389). In light of this, we call on those designing health interventions to foreground the importance of creating shared ‘timespaces’ (Schatzki, 2009) that flex and extend beyond the boundaries of linearity. Doing so would increase the possibilities for interventions (and related activities) to coincide and foster harmonious connections with the ever‐changing lived realities of the people they intend to support.

As such, we suggest that rather than imposing hegemonic intervention time, interventions must ensure that intervention timespaces persistently coincide with those of its clients. Rather than requiring clients to create and maintain synchronised temporal connections with the ‘linear causal chains’ (Orton et al., 2017, p. 478) often characterising health interventions, we suggest actively acknowledging and attending to the competing practices which constrain engagement. This means allocating resources to foster close, embedded connections which are reactive and responsive. In sum, we argue that social prescribing, and other interventions offering ‘personalised support’, should make persistent efforts to synchronise with the heterogeneous tempos and temporal rhythms of the people they seek to support.

## AUTHOR CONTRIBUTION

**Kate Gibson:** Conceptualization; Lead, Data curation; Lead, Formal analysis; Lead, Investigation; Lead, Writing – original draft; Lead, **Suzanne Moffatt:** Conceptualization; Supporting, Formal analysis; Supporting, Funding acquisition; Lead, Investigation; Supporting, Methodology; Supporting, Supervision; Equal, Writing – review & editing; Equal, **Tessa Pollard:** Conceptualization; Supporting, Formal analysis; Supporting, Funding acquisition; Supporting, Investigation; Supporting, Methodology; Lead, Supervision; Equal, Writing – review & editing; Lead.

## Data Availability

The data that support the findings of this study are available on request from the corresponding author. The data are not publicly available due to privacy or ethical restrictions.

## ENDNOTES

^1^
In the UK, benefits claimants who are deemed to have not met certain conditions are subject to a benefits sanction by the Department of Work and Pensions (DWP) who will reduce or stop a claimants' benefits.

^2^
Medical assessments are undertaken by the DWP with those who are unable to work due to health issues to assess the validity of their benefit entitlement. This process is lengthy, subject to delay, and claimants who are unsuccessful in this process are required to navigate a complex appeal process.
